# A micro-fabricated *in vitro* complex neuronal circuit platform

**DOI:** 10.1088/2057-1976/ab2307

**Published:** 2019-06-03

**Authors:** M Kamudzandu, M Köse-Dunn, M G Evans, R A Fricker, P Roach

**Affiliations:** 1 School of Medicine/Institute for Science and Technology in Medicine, Keele University, Staffordshire. ST5 5BG. United Kingdom; 2 School of Life Sciences, Keele University, Staffordshire. ST5 5BG. United Kingdom; 3 Department of Chemistry, School of Science, Loughborough University, Loughborough, Leicestershire. LE11 3TU. United Kingdom; p.roach@lboro.ac.uk

**Keywords:** *in vitro* model, neuronal network, basal ganglia, microfluidic, 3Rs, *brain-on-a-chip*

## Abstract

Developments in micro-manufacture as well as biofabrication technologies are driving our ability to create complex tissue models such as ‘*organ-on-a-chip*’ devices. The complexity of neural tissue, however, requires precisely specific cellular connectivity across many neuronal populations, and thus there have been limited reports of complex ‘*brain-on-a-chip*’ technologies modelling specific cellular circuit function. Here we describe the development of a model of *in vitro* brain circuitry designed to accurately reproduce part of the complex circuitry involved in neurodegenerative diseases; using segregated co-culture of specific basal ganglia (BG) neuronal subtypes to model central nervous system circuitry. Lithographic methods and chemical modification were used to form structured micro-channels, which were populated by specifically cultured neuronal sub-types to represent parts of the inter-communicating neural circuit. Cell morphological assessment and immunostaining showed connectivity, which was supported by electrophysiology measurements. Electrical activity of cells was measured using patch-clamp, showing voltage dependant Na^+^ and K^+^ currents, and blocking of Na^+^ current by TTX, and calcium imaging showing TTX-sensitive slow Ca^2+^ oscillations resulting from action potentials. Monitoring cells across connected ports post-TTX addition demonstrated both upstream and downstream changes in activity, indicating network connectivity. The model developed herein provides a platform technology that could be used to better understand neurological function and dysfunction, contributing to a growing urgency for better treatments of neurodegenerative disease. We anticipate the use of this advancing technology for the assessment of pharmaceutical and cellular therapies as a means of pre-clinical assessment, and further for the advancement of neural engineering approaches for tissue engineering.

## 1. Introduction

Micro-circuits of the central nervous system (CNS) consist of complex networks of neurons interconnected via intricate and extensive processes. The basal ganglia (BG) micro-circuit consists of discrete nuclei located in distinct regions or compartments of the deep brain [1, 2]. Building compartmentalized systems that closely mimic the architecture of neuronal populations in the CNS provides the means to study the interaction and connectivity of neurons in these highly complex networks. Moreover, such *in vitro* systems provide the means necessary to accurately fabricate much-needed models for screening potential neurodegenerative disease therapies [3–5]. In addition, advances in micro-fabrication techniques, either inspired by established procedures or those used to manufacture integrated circuits in the semiconductor industry [6], allow us to advance our ability to create multi-cellular constructs to a stage where organ on a chip terminology is relativity commonplace within the biomaterials and tissue engineering communities. These models encompass many different systems, although the complexity of the CNS remains a challenge [7, 8]. This is, in part, due to our lack of knowledge of the interconnectivity of such complex systems, having a plethora of different cell types intercommunicating across multiple regions of the brain simultaneously.

Current state-of-the-art animal and tissue CNS models are limited in their ability to give characteristic information on specific cell input/activity within discrete circuits, and certainly these models have huge limitations ethically and come at huge financial cost. *In vitro* models allow for: (1) segregated co culture of cells, (2) tighter control over cell manipulation, (3) enhanced ability for cell observation and measurement, (4) control over directed cell connectivity using the designed structure of the device. Devices presenting internalised channels that guide axonal outgrowth, either termed microfluidic or more precisely micro-structured (as flow in microfluidic systems can be damaging to sensitive neuronal cells), have been of interest for the reconstruction of neuronal circuitry [9]. Reports of using materials presenting pores for the construction of layered semi-organised networks have also shown promise [10]. The most advanced micro-structured systems report the culture of only a few different cell types, with limited ability to biofabricate designer neuronal circuits [11–13]. The segregated co-culture of compartmentalised neurons is a well-established method to study cell-cell connectivity, e.g. neuron neuron or neuron-glia, yet there are few reports on the construction of larger, more representative cell circuit models, with these being of central interest in disease pathways yet to be studied [14–18].

*‘Brain-on-a-chip*’ devices have been established for the investigation of neurodegenerative diseases [19–21]. Kunze *et al* (2011) fabricated an *in vitro* Alzheimer’s disease model to study the neurodegeneration process as well as to screen for potential therapies [20]. A simplified model separating two populations of primary cortical neurons by micro-channels was presented, with okadaic acid being used to selectively induce neurotoxicity in one population, whilst the effects were studied across the neuronal network. Micro-fabrication methods have been investigated to better control directionality of axonal connectivity, and therefore achieve more robust network engineering. Peyrin *et al* (2011) presented tapered micro-channels to act as ‘axonal diodes’ [5], with more recent advances using curved interconnected channels to limit unwanted connections in one direction [22]. Others have reported the use of a vacuum to induce tension-directed neurite growth in micro-channels [23]. Importantly, these devices have not been used to engineer large multi-cellular complex neuronal circuits, and have therefore not been adopted by the neuroscience community as much as they could be. This community is actively seeking new tools and technologies to advance the understanding of brain function and connectivity.

Here we report the fabrication of a unique five-port microfluidic device to provide an *in vitro* functional model that mimics the *in vivo* BG circuit, whose degeneration leads to Huntington’s disease (HD) and Parkinson’s disease (PD) pathologies. We set out to re-establish the BG circuit *in vitro* with sub-types of neurons dissociated from the rat brain cultured separately in a five-port micro-channel presenting device produced by soft lithography. Directional connectivity was established via tapered micro-channel geometry to specifically engineer CNS regional connectivity. Functional connectivity was assessed using patch clamp, and calcium imaging within each neuronal sub population. The methodology reported here will potentially provide the means to better understand normal BG function and progression of neurodegenerative disease mechanisms such as in HD and PD, as well as serving as a more realistic *in vitro* model. This will enable more informed pharmaceutical and cellular therapies to screen for informed pre-clinical evaluation, and as such can be used as a powerful tissue engineering platform.

## 2. Materials and methods

All chemicals were used as received unless otherwise stated.

### 2.1. Micro-device fabrication

**Figure 1.**
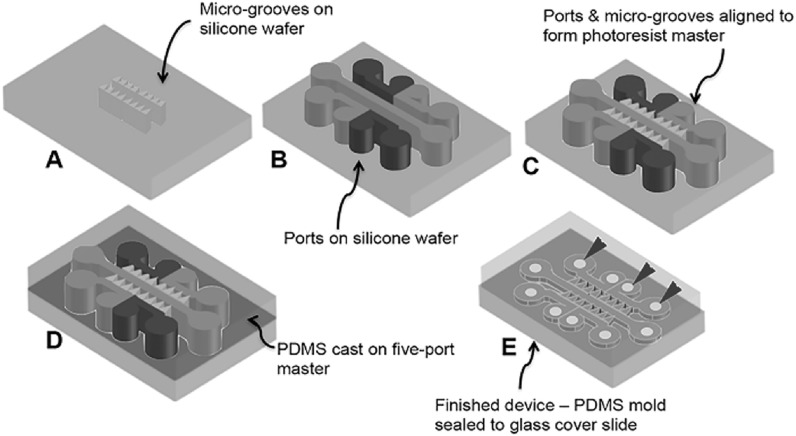
Schematic showing fabrication of the microfluidic device. (A)–(C): Photolithography was used to fabricate the master. (D)–(E): Soft lithography was used to inversely replicate the master and produce the devices. Holes indicated by blue arrowheads in E allowed access to microfluidic devices and were drilled into PDMS moulds before sealing the devices to glass cover slides.

Devices were designed in AutoCAD to produce two chrome-glass masks presenting separately millimetre sized ‘culture ports’ and smaller micro-channels. Conventional photolithography was used to prepare master devices in SU8 on silicon wafers, figure 1. SU8-10 (MicroChem) was used for the micro-channel structures, being spun onto a silicon wafer at 4000 rpm for 30 s; pre-baked at 65 °C for 1 min and then 95 °C for 5 min. Exposure was carried out at i-line using a Canon PLA-501 set at ∼90 mJcm^−1^. After developing, a layer of SU8-50 (MicroChem) was spun atop at 3000 rpm, pre baking at 65 °C for 5 min and then 95 °C for 30 min. After developing and drying, this template was exposed to chlorotrimethyoxysilane (Sigma) vapour at ∼0.3 atm overnight. This was sufficient to give a coating of sufficient hydrophobicity to reduce adherence of polydimethylsiloxane (PDMS) during the next stage. Soft lithography replication of the master was performed using PDMS (Sylgard 184; 10:1 polymer:cross-linker), set by heating at 70 °C for 45 min. PDMS devices were chemically bonded to a glass base using oxygen plasma etching, giving rise to an increased wettability within the device channels, using a Deiner instrument O_2_ ∼ 0.3 mbar, 30W, 13.56 MHz RF plasma for 30 s. This treatment left inner surfaces of the channels hydrophilic such that virtually no pressure was required to introduce fluids such as phosphate buffered saline (PBS, Sigma), poly-D-lysine (PDL, Sigma) and laminin solutions (Sigma), as well as neuronal culture medium, into the device.

### 2.2. Tissue extraction and cell suspension preparation

Pregnant Sprague-Dawley rats and their embryos were bred in-house at Keele University with tissues obtained following approved Schedule 1 methods from the UK Animals Scientific Procedures Act 1986 and authorization from Keele University’s local ethics committee. The embryos were either 15-16 days old (E15-E16) or 12 13 days old (E12-13), with E0 defined as date of observing vaginal plug. Cortical, lateral ganglionic eminence (LGE), medial ganglionic eminence (MGE) and ventral mesencephalon (VM) brain tissue were dissected out. Substantia nigra (SN), globus pallidal (GP) and striatal neurons develop from the VM, MGE and the LGE, respectively. Tissue pieces were placed into a 1.5 ml Eppendorf containing approximately 1 ml of Dulbecco’s Modified Eagle’s Medium/Nutrient F-12 Ham’s medium (DMEM-F12, Sigma Aldrich, Dorset, United Kingdom). Tissue pieces were washed twice with DMEM-F12 and placed into a mixture of 0.1% trypsin (Worthington Biochemical Corp., Reading, UK) and 0.05% DNase (Greiner Bio-one, UK) in DMEM-F12 for 20 min. The pieces were then washed three times with DNase solution before they were mechanically triturated into individual neurons. The neurons were centrifuged at 160 RCF for 3 min. The supernatant was aspirated out and neurons were re-suspended in neuronal culture medium. Dissociated neurons were counted using a haemocytometer (Fisher Scientific, Loughborough, UK) and their viability was checked using trypan blue (Invitrogen, Paisley, UK) before cell culture. Neurons were cultured in neuronal culture medium (NCM), which consisted of: 95% neurobasal, 1% fetal calf serum (FCS), 1% penicillin/streptomycin/fungizone (PSF), 1% B27 supplement (all from Invitrogen, Paisley, UK), 10 mM L glutamine (PAA, UK) and 0.45% glucose (Sigma-Aldrich, Dorset, United Kingdom). Seeding density of 10,000 to 20,000 neurons *μ*l^−1^ was prepared in NCM.

### 2.3. Microfluidic device cell loading and culture

NCM was aspirated from reservoirs but not from channels (to avoid bubbles) and ports were seeded with ∼20,000 cells *μ*l^−1^ in 10-15 *μ*l. Cells were incubated for ∼2 h at 37 °C to allow them to attach. Thereafter, devices were filled with 150–200 *μ*l of NCM per port and were placed in the incubator and monitored for 12-18 days. NCM was replaced every 2 to 3 days.

### 2.4. Functional characterisation

#### 2.4.1. Patch clamp

Whole cell patch clamp recordings were obtained from E15 primary cortical neurons cultured for 14–18 days *in vitro*. Recordings were done on the stage of an upright microscope fitted with a ×40 water-immersion objective lens (Olympus BX51). The intracellular solution consisted of (mM): KCl 140, MgCl_2_ 3, EGTA 1, HEPES 5, NaGTP 0.3, pH 7.3 with KOH. Bath solution was NCM. Glass micro-pipettes had a resistance of 3–4 ΜΩ when filled. The pipette shanks were coated with ski wax (Toko AG, Switzerland) to reduce capacitance. Cells cultured on a coverslip were placed in the recording chamber and secured using a small drop of Sylgard polymer (Dow Corning). Whole-cell recordings were made using a List EPC-7 amplifier (HEKA, Germany). TTX (5 *μ*M) was dissolved in the external solution and ejected from second pipette by gas pressure controlled through solenoid valves (WPI picopump). The duration of the application was 10 s. The pipette was positioned to within about 10 *μ*m of the cell.

#### 2.4.2. Calcium imaging

Devices with striatal, cortical, SN and GP neurons at DIV 14-18 were loaded with 2 *μ*M Fluo-4 AM (Ca^2+^ indicator dye). Neurons were incubated in the dye for 30 min at room temperature before washing with loading buffer (150 mM NaCl, 4 mM KCl, 1 mM MgCl_2_, 10 mM glucose, 2 mM CaCl_2_ and 10 mM HEPES dissolved in sterile distilled water) and replaced with DMEM-F12. Ca^2+^ fluorescence oscillations were captured on a Nikon Eclipse 80i microscope, using 480/30:535/45 ex/em filters. 1 *μ*M of tetrodotoxin (TTX) in

NCM was added to cortex, striatum or GP ports for microfluidic devices. Pre-TTX and post-TTX recordings were simultaneously acquired at 5 fps for 3 min for two adjacent ports, using a 4 × objective lens to obtain a population image allowing multiple cells to be observed in one region of interest, with ∼2 min between each recording. Ca^2+^ fluorescence intensity recordings were obtained from individual cells using ImageJ. Changes in fluorescence intensity are reported relative to background fluorescence (non-cell region of interest showing degree of non-specific background fluorescence) (F/F_0_). Data is shown for the investigation across one single device although this was repeated thrice; the data shown is representative of all three repeats.

### 2.5. Fluorescence imaging

To check for region-specificity, 100,000 cells of each regional dissection were cultured for 8 DIV and stained for region-specific neuronal markers (n = 4). Cultures were fixed with 4% paraformaldehyde (PFA) (Sigma) before staining with fluorescent antibodies. Media was removed and cells washed 3 times with tris-buffered saline (TBS) (12 g trizma base from MERCK, Germany, with 9 g NaCl in 1 L dH2O) then blocked and permeablised with 5% normal goat serum (NGS) and triton-X (1:500) in solution with TBS for one hour at 4 °C. The primary antibody solutions consisted of TBS with 1% NGS, 1:500 triton-X and the selected antibody: *β*-tubulin III (mouse; 1:1000), Gamma-aminobutyric acid (GABA; rabbit; 1:500), vesicular glutamate transporter II (VGLUT2; mouse; 1:500), phosphotyrosine (PY; mouse; 1:500). Cultures were then washed 3 times in TBS before addition of secondary antibodies: Alexa Fluor 488 (goat anti-rabbit, red fluorescence) or Alexa Fluor 555 (goat anti-mouse, green fluorescence), both at a dilution ratio of 1:300, for 2 h at room temperature in the dark. Cultures were then washed 3 times with TBS before being mounted onto microscope slides using Vestashield softset mounting medium with 4′,6-diamidino-2-phenylindole (DAPI) (Vector laboratories). Images were collected on a Nikon Eclipse 80i microscope.

## 3. Results

### 3.1. Cell laden devices for neural circuit construction

**Figure 2.**
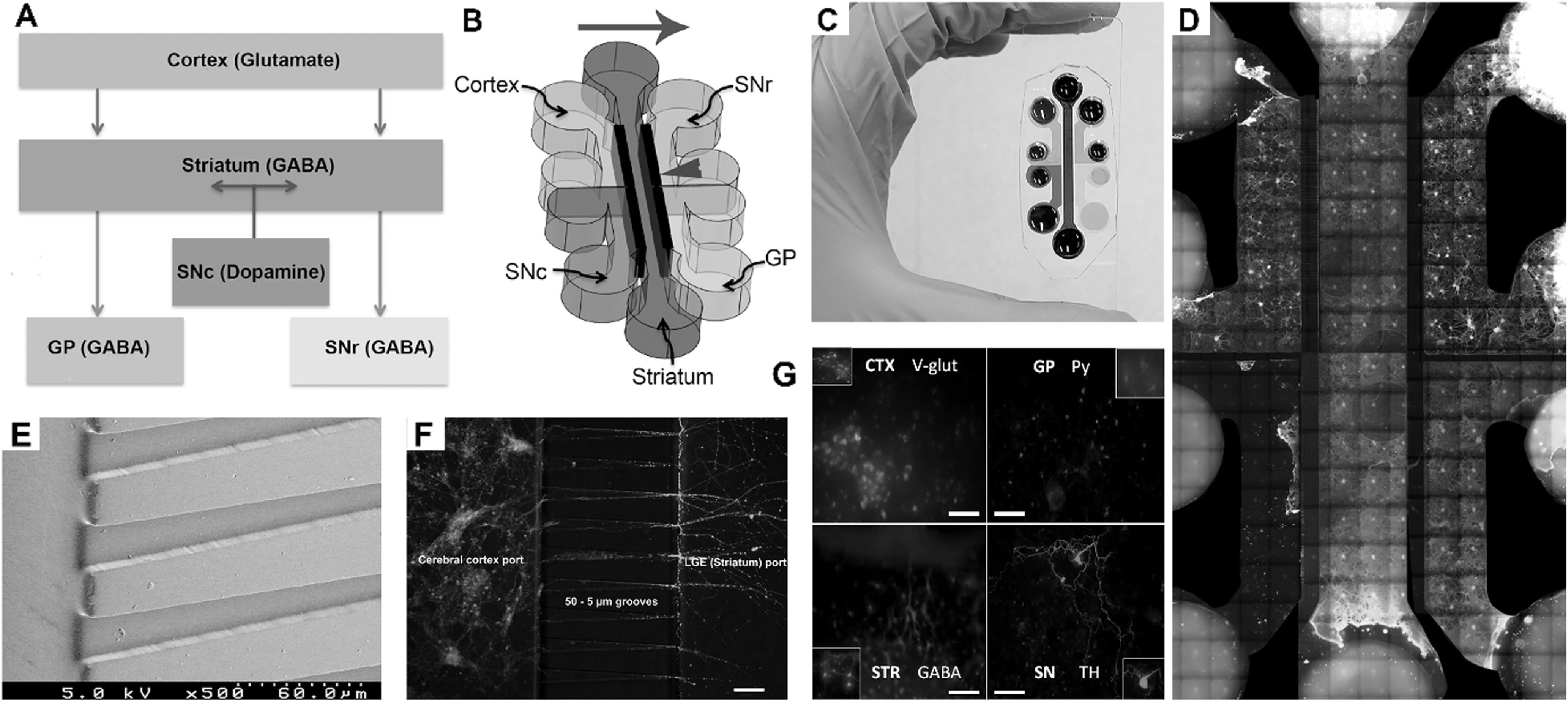
The BG neuron circuitry *in vitro* model. (A) Schematic of the BG circuit. Nuclei connectivity is regulated by neurotransmitters and established via axons (arrows). Red arrows = inhibitory GABAergic, green = excitatory glutamatergic, and blue = dopaminergic impulse transmission. (B) Five-port microfluidic device designed to model key components of the BG circuit seen in A. Tapered micro-channels were used to control direction of axon orientation, shown by the blue arrowhead. Brown arrow indicates direction of channel tapering/axon orientation, and the bold black parallel lines indicate the position of the micro-channels. (C) PDMS microfluidic mould fabricated via soft lithography. Coloured dye indicating the separation of fluids between ports with colours corresponding to the arrangement of neuronal types as presented in (A) and (B). (D) Whole cell-seeded device with DAPI and *β*-III-tubulin staining as blue and green respectively. (E) SEM micrograph of tapered micro-channels (whole channel not shown). (F) Fluorescent image of antibody stained neurons (green, *β*-III-tubulin) and astrocytes (red, GFAP) on either side of 50-5 *μ*m tapered micro-channels. The neurons extend from the cortical port into the striatal port, similarly to their activity *in vivo*. (G): Antibody characterisation of neuronal sub-types, with cortex, STR (striatum), GP and SN stained with V-GLUT2, GABA, PY and TH antibodies respectively.

The soft lithography process enabled multiple neuronal cell types to be collectively cultured together in a single device, only able to communicate only via extension of axons through micro-channels connecting the culture ports in a specific geometry. Our design was inspired by the arrangement of neuronal populations in a well-established BG circuit [23], figure 2(A). The microfluidic device consisted of one long port in the middle to house striatal neurons, flanked by two ports on each side to house neurons from the two input regions to the striatum and two main output targets of the striatal neurons, figure 2(B). Contact guidance provides alignment to axons [23], therefore enabling controlled projection from one compartment to another, and the length of micro channels can prevent shorter dendrites from traversing. Consequently, isolated axons can be analysed separately without interference from the soma and dendrites, e.g. for axonal growth, injury or degeneration studies. Using an aqueous dye we were quickly able to see the separation of fluids in each of the ports as seen in figure 2(C), with the micro-channel areas showing very little diffusion mixing of the solutions even after an hour due to the small cross-sectional area and extended length of the channels, figure 2(E). Since these experiments were conducted in cell-free devices, the ability of cell media to diffuse between ports when neurites were growing through the channels was considered to be extremely limited. This was particularly relevant in later experiments when toxins were selectively introduced. An array image of the whole device is shown in figure 2(D) with magnified cortex-striatal microchannel region shown in figure 2(F). Validation of each of the neuronal sub-types was fully characterised via fluorescence labelling, figure 2(G).

### 3.2. Cell culture in micro-devices

**Figure 3.**
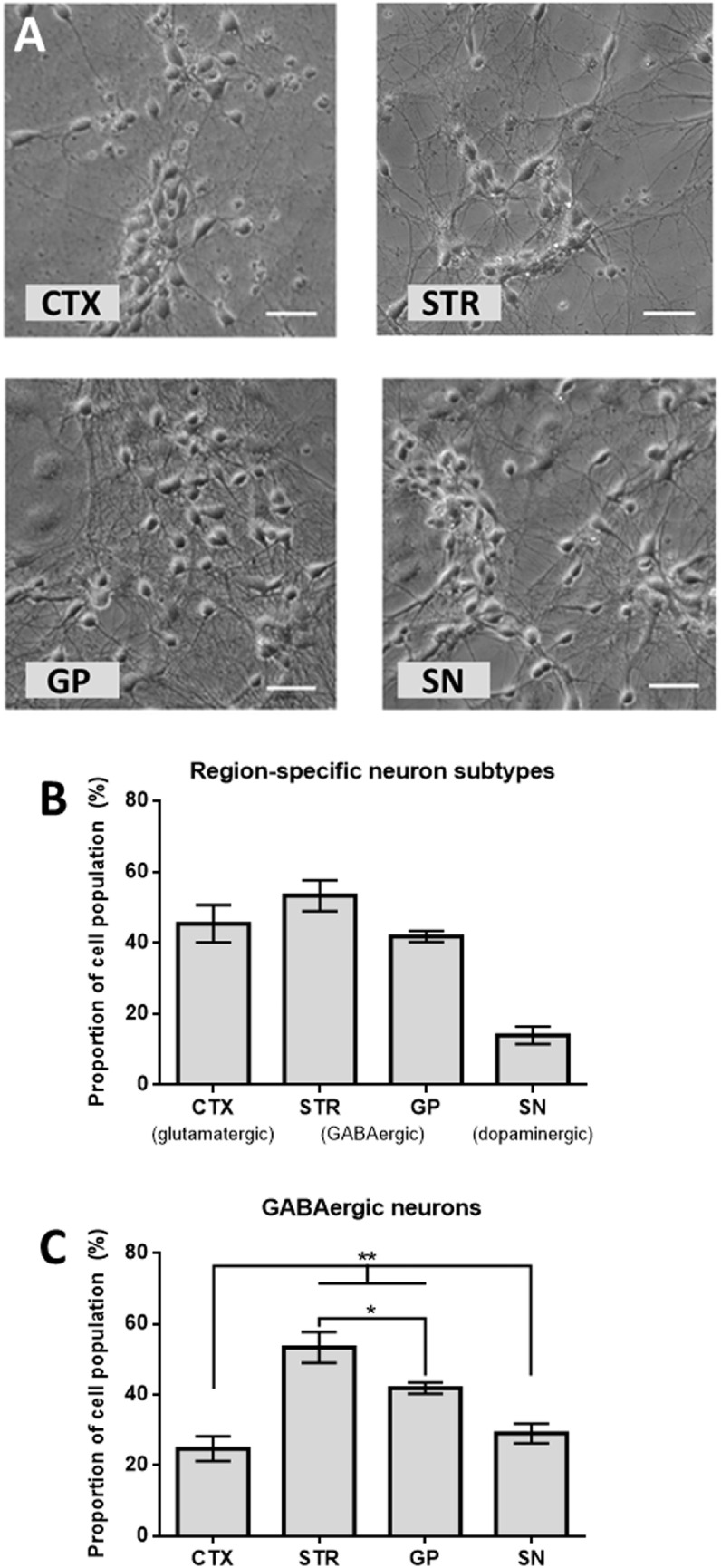
Cells within our devices. (A) Brightfield images of cortical (CTX), striatal (STR), GP and SN cells within a microfluidic device after 9 DIV, representative of all ports observed. Scale bars = 20 *μ*m. (B) The proportion of cells in each specific port that stained positive for the region-specific neuronal subtype (in brackets) marker (n = 4). (C) The proportions of cells in each specific port that stained positive for GABA neuronal marker (n = 4). Asterisks represent post-hoc t-tests, (*): p < 0.05, (**): p < 0.01.

Biological pre-conditioning of PDMS or glass is often carried out for the culture of cells in micro devices. This is particularly relevant for sensitive neural cells, e.g. poly-D-lysine (PDL) and polyornithine and/or extracellular matrix (ECM) proteins are often used to promote growth and development of processes [23]. Here we found PDL/laminin coatings within the culture ports and micro-channels worked well to support attachment and outgrowth of primary neural sub-types. Cultures of healthy phase-bright neurons were observed both within control cultures on open coverslips in 24 well plates, and in cultures within the micro-devices over the 18 DIV experiment (figure 3(A)). Due to cell clustering within cultures, and a limited number of ports and devices for analysis, total cell numbers within each port could not be obtained. However, efficient seeding of cells led to robust populations of neurons with mature morphologies within all cultures, up to the 18 days investigated (figure 3(A)).

Immunofluorescence labeling of glutamatergic, GABAergic and dopaminergic neurons (figure 2(G)), identified appropriate proportions of regional-specific neurons within each specific port, figure 3(B). All ports contained GABAergic neurons, and there were signifcant differences in the proportion of GABAergic neurons generated from each brain region (ANOVA (F_(3,8)_ = 48.45, p < 0.001; figure 3(C)). As expected, STR and GP ports contained significantly higher levels of GABAergic neurons when compared to CTX and SN. There were significantly more GABAergic neurons in the STR port than the GP port and no difference in the number of GABAergic neurons when comparing the CTX and SN ports. After 7 days in culture axonal projections were clearly visible, being guided within micro-channels. By day 14 long axonal extensions were identified spanning between culture ports, sprouting from the narrow end of tapered channels on exit, as seen in figure 2(F) and supplementary figure 1. Cortical, striatal and SN neurons all showed robust extension of axons across the microchannels exiting to the adjacent port. Varying combinations of channel entrance and exit widths were tested with no striking differences evident when comparing neurites exiting different channel structures, therefore data were combined.

### 3.3. Functional assessment of neuronal circuitry

#### 3.3.1. Patch clamp

Whole-cell recording (patch-clamping) demonstrated electrical activity of E16 cortical or striatal neurons cultured on glass coverslips, with inward Na^+^ currents, and outward K^+^ currents, being generated in response to depolarising voltage clamp steps (supplementary information figure 2(A)). The I–V curve is also shown (supplementary figure 2(C)), measured at early and late times to indicate both currents (as indicated by the arrows in figure 3(A)). The Na^+^ current was completely blocked by TTX (supplementary figure 2(b)). Voltage-gated Na^+^ current initiates generation of action potentials in excitable cells. In current clamp, it was found that the neurons possessed spontaneous and depolarisation-induced spiking activity, indicating the excitability of these neurons at this early stage. Voltage records are shown in supplementary figure 2(D) demonstrating evoked action potentials during the depolarising current steps and occasionally spontaneous action potentials afterwards.

#### 3.3.2. Calcium imaging

Calcium oscillations were measured in specific neuronal populations cultured within our device, both before and after the application of tetrodotoxin (TTX). This is a neurotoxin that blocks sodium voltage gated ion channels and hence prevents neurons from firing action potentials. TTX was introduced separately to cortex, striatum or GP ports (via pipette into specific port of interest) to determine the functionality and connectivity of the BG neurons, in order to investigate the responses of neuronal populations belonging to upstream and downstream ports within the microfluidic device. The terms ‘upstream’ and ‘downstream’ refer to the cellular geometry of input neurons and targeted neuronal outputs.

To check that TTX was not able to diffuse across the micro-channels, a fluorescent dye diffusion assay was performed using rhodamine and fluorescein isothiocyanate (FITC), fluorescent dyes of similar molecular mass to TTX. Results showed that the dyes diffused very slowly into micro-channels, and dye molecules had only travelled one third of the distance through cell-free channels by 30 min (Supplementary figure 3). All Ca^2+^ measurements were undertaken within 20 min of addition of TTX using conventional approaches [23]. Ca^2+^ oscillations were recorded from individual neurons cultured within each of the five neuronal populations, before and after TTX was applied specifically to different ports.

**Figure 4.**
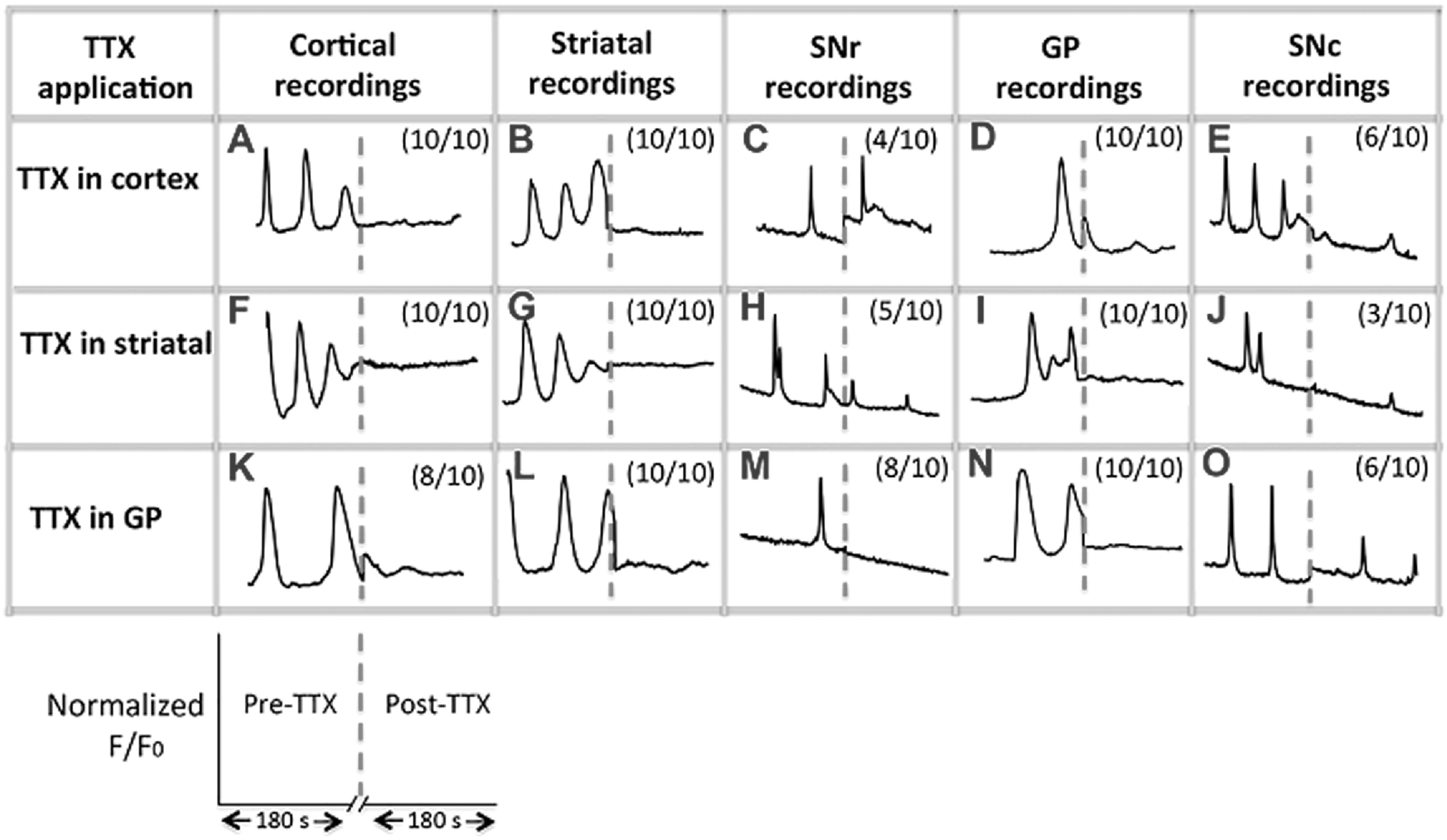
Calcium oscillations showing representative waveforms across all connected cell types. Recordings were carried out from individual cells in networks within one device, before and after addition of TTX (red dashed line). Numbers in brackets indicate how many of the 10 neurons recorded in each port showed the presented response, with the remainder not being dampened to the same extent by TTX addition. Cell activity was recorded for 180 s before and after TTX. F is the recorded fluorescence intensity and F_0_ the initial background fluorescence. Normalized F/F_0_ y-axes are shown at maximum scale for ease of qualitative visualization.

The effects of the toxin on directly affected cells as well as upstream and downstream effects were recorded. Ten oscillating neurons were selected for observation from each group; this number being limited by the time constraints of recording from all connected populations within a single device. It was of major importance to understand how the action of TTX on one cell type affected the whole network activity. Data is reported in terms of representative fluorescence oscillations from each group, figure 4. Cells that were considered outside of this representative response showed opposite responses post-TTX addition, e.g. if they are shown in figure 4 to present no spiking post-TTX addition, the opposite trend would be continued spiking activity. In general, all cell populations showed calcium signalling oscillations before the application of TTX. All populations to which TTX was directly applied showed a marked decrease in activity. Those cells connected only via the cellular network in the device, i.e. not present in the port where TTX was applied, also showed a rapid change in response. Some cells, particularly those within SNr, showed continued activity after TTX administration in either cortical, or striatal ports.

Activity within the microfluidic devices was observed to be similar to neuronal activity in isolated cultures. Cortical neurons produced spontaneous activity, indicated by Ca^2+^ oscillations, and this was abolished by direct application of TTX onto these cells (figure 4(A)). This response was observed in all neurons measured (10/10) indicating excellent correlation of activity across the cortical network and is supported by single patch clamp recordings, figure 3. Striatal neurons also yielded Ca^2+^ oscillations that were abolished by addition of TTX to the striatal port (figure 4(F)) demonstrating that this activity was most likely the firing of action potentials (again supported by patch clamp experiments (figure S2)). GP neurons were also TTX-sensitive: spontaneous Ca^2+^ oscillations were present in untreated cultures, but were eliminated by TTX (figure 4(K)), suggesting that they possessed spontaneous spiking activity that can be modulated with a neurotoxin.

## 4. Discussion

Fabricated micro-structured devices have demonstrated potential for neural bioengineering; offering the ability to form segregated co-cultures of discrete neuronal populations that are linked via axons connecting through micro-channels [7]. Here we have shown successful culture to excitable maturity of multiple different primary neuronal sub-types and their axonal outgrowth to adjacent ports within a 5-port microfluidic device in order to work as a simple neuronal circuit.

### 4.1. Multiple population connectivity

Biofabrication of complex neural cell circuitry has been investigated, with specific focus on the functional connectivity, which is of the utmost importance for neuronal models. This work builds upon previous reports which have mostly limited their focus to the controlled connectivity of two neuronal populations via the use of channels, [9, 11, 24–26] grooves [24, 27] or fibres [27, 28]. Many reports in the area of micro-fabricated neuronal networks aim to define a methodology of neuron-neuron network formation [29] rather than to fabricate a model which specifically mimics connectivity across multiple brain regions. Others have demonstarted the exquisite ability to control placement of two populations onto a multi-electrode array, and postulate that similar methods can be used for the localised positioning of up to 4 discrete cell populations. [30] These technologies are advancing our ability to fabricate defined regional specific and closely seeded neuronal sub-types; our work has advanced this by combining multiple cell types with controlled directional connectivity using tapered channels, demonstrating functionally connected regions.

Controlled *in vitro*, biofabrication requires precise use of cell types to construct realistic mimics of the required tissues—in this case a specific cellular circuit of the CNS. In previous reports of neural circuit formation, using only a limited number of different cell types, the source of cells is often considered yet there are few reports which consider regional connectivity of primary CNS neuronal sub-types. Peyrin *et al* (2011) demonstrated the use of tapered micro-channel connectivity within a two-port microfluidic device for the fabrication and characterizion a cortico-striatal oriented network, [5] which was a major advance in CNS modelling *in vitro*.

Here we present a methodology to construct a specific model of advanced CNS neuronal cell circuitry which is central to understanding normal and diseased function for, e.g. Parkinson’s disease. Working from an engineering basis the discrete cellular types required were harvested from a primary rodent source; a schematic representing these populations and how they were cultured adjacent to each other is shown in figure 2. Our assessment of the each of these discrete populations clearly demonstrated a healthy neuronal population in each port (figure 3), good attachment, continued outgrowth and directed neurite elongation within the micro-devices over the 18 days investigated.

All populations were found to culture well over long periods in the same media, with optimisation of surface chemical coatings allowing control *over* adhesion and neurite outgrowth. The micro-channel walls, acting in synergy with the PDL/laminin surface presented, gave rise to good contact guidance cues for neurites.

### 4.2. Functional neuronal connectivity

Morphologically, cultured neurons extended axons through microchannels exiting in the adjacent ports, yet functional assessment is key to understand the absolute fabrication of such a complex network. We first confirmed neuronal activity of dissected neurons using patch clamp electrophysiology; with voltage clamp to identify inward Na^+^ currents and spiking activity, supplementary information, figure S2. Cultured E16 cortical neurons showed spontaneous and depolarisation-induced spiking activity. Cortical neurons represent the main excitatory input to the basal ganglia [31–33], being of significant electrical importance to this model cell circuitry. The electrical activity observed in cortical neurons in the current study aligned well to that previously observed in primary cortical cultures [34]. Therefore, we were confident that our primary neurons would possess appropriate electrical firing properties when cultured in microfluidic devices.

Using patch clamp methodology, we directly recorded action potentials from neurons also presenting voltage-sensitive Na^+^ currents (e.g. associated with spiking) and demonstrated that these Na^+^ currents were blocked by TTX, supplementary information, figure S2(B). Patch clamp recordings could not be made within a device due to geometry constraints.

Calcium imaging was used to extend monitoring of cells across each population and across the device in real-time. This method enabled us to simultaneously record wave-type spiking, assumed to be linked to the action potential spiking observed in patch clamp, across the whole device at single-cell resolution [35, 36]. Manual imaging enabled identification of active neurons connected in networks across all the populations in a single device. However, to do this only a limited number of cells could be imaged within each port. Ca^2+^ oscillations were studied in BG neurons as unconnected circuits for cortical and striatal neurons (cultured as individual populations; data not shown), and connected neurons (cultured in five-port connected network devices). Thus, this method was chosen to study the responses of populations of neurons in real-time: as isolated sub-types, in connectivity with the microfluidic devices, and following application of the neurotoxin TTX to individual ports within a device.

### 4.3. Reduced neuronal activity downstream of TTX treatment

Downstream neurons showed reduced activity in all cases when TTX was administered to upstream neurons. We confirmed that there was no transfer of TTX to the downstream ports within the timescale of the experiments, both directly through a dye diffusion study (supplementary information, figure S3) and indirectly through the continued presence of Ca^2+^ oscillations in other ports (discussed below). Evidence from brightfield and fluorescence imaging suggested that axons had extended into downstream ports, for example cortical axons were observed within striatal ports by DIV 12-13 (figure 2(F), supplementary information, figure S1 is available online at stacks.iop.org/BPEX/5/045016/mmedia). Therefore, any reduction in neuronal activity downstream of TTX administration was hypothesised to be due to inter-neuronal connectivity.

All neurons in ports downstream from where TTX had been administered showed a reduction in spontaneous spiking. Striatal neurons yielded Ca^2+^ oscillations that were abolished after TTX was loaded into the cortex port (figure 4(B)). This response observed in all 10 neurons examined strongly suggests the functional connectivity of the cortical and striatal neuronal populations. Cortical neurons are excitatory and are known to excite inactive striatal neurons in dissociated mixed cultures [37] and have also shown this repsonse when cultured in a two-port micro fluidic device [5].

There was a reduction in GP (figure 4(D)) and SNr activity after TTX application to the cortical port. Cortical neurons should not normally be directly connected to either GP or SNr neurons; connection *in vivo* is indirect via the striatum. As *in vivo*, our device was designed so that the GP and SNr ports were separated from the cortical port by the striatal port. The response of GP and SNr neurons to the silencing of cortical neurons suggests that there was communication between the neuronal populations, with striatal neurons bridging them. Interestingly, some small residual Ca^2+^ oscillations were observed in 4/10 SNr neurons after TTX was applied to the cortical port, figure 4(C). A reduction in activity was observed in SNr (figure 4(H)) and GP (figure 4(I)) activity after TTX application to the striatal port, suggesting that functional connectivity had indeed been established across all populations in the 5-port model.

Small residual oscillation traces in 5/10 neurons after TTX likely represent dopamine neurons that are developing in the closely apposed SNc tissue, which are likely present in the dissected cells. A number of studies have reported the pacemaker-like, spontaneous firing nature of SNc dopamine neurons [38], with characteristic electrophysiology behaviour *in vitro* [33] that may have been unaffected by TTX application.

Since the striatum is GABAergic and therefore inhibitory, the output response of GP neurons in response to TTX application in the striatal port should in theory be increased firing. However, it is known that immature GABAergic neurons (such as striatal neurons) are excitatory in nature [39] as the GABA neurotransmitter can be excitatory during early development. Immature CNS neurons express more of the Na^+^-K^+^-2CI^−^ (NKCC1) co-transporter than the K^+^-CI^−^ (KCC2) co-transporter, leading to increased intracellular chloride concentration promoting excitatory postsynaptic potentials rather than inhibitory postsynaptic potentials. Mature neurons express more KCC2 than NKCC1, leading to membrane hyperpolarization [40, 41]. Therefore, it is possible that the reduction of Ca^2+^ oscillations in downstream GP neurons was simply a direct response to elimination of excitatory activity in the striatal neurons.

### 4.4. Reduced neuronal activity upstream of TTX treatment

Similarly to downstream ports, all neurons within ports upstream of ports where TTX was administered showed a reduction in Ca^2+^ oscillations. Cortical neurons showed immediate and significant reduced activity after the treatment of striatal neurons with TTX, as seen in figure 4(F). This provided further evidence that connectivity was established between cortical and striatal neurons in the device. Other studies using slice culture techniques have also shown that changes in striatal neuron activity can affect changes in the activity of cortical inputs, for instance in a mouse R6/2 HD model [42], where the frequency of spontaneous excitatory postsynaptic currents (EPSCs) generated from the cortex and transmitted into the striatum deteriorated as HD symptoms manifested.

Striatal neurons showed significantly reduced activity after the treatment of GP neurons with TTX, as seen in figure 4(L). Abolishing action potential activity in GP neurons resulted in significant reduction of Ca^2+^ oscillations in striatal neurons. This suggests that striatal and GP neurons had formed a functional connection. We aimed to fabricate devices in which only the axons from striatal neurons innervated the GP port, however there is a small probability that the GP neurons could have extended axons back into the striatal port. There is *in vivo* evidence of a sub population of GABAergic GP neurons that can project their axons back to the striatum [42], and therefore have significant control over the activity of striatal neurons [2]. If the latter had occurred, these GP neurons could have influenced striatal spiking activity [41].

After treatment of GP neurons with TTX there were also indirect changes in the activity of neurons in all other parts of the circuit. The response of cortical neurons suggests there is networked communication between cortical and GP neurons. This communication is likely via striatal neurons rather than directly integrating GP neurons with cortical neurons, due to the distances involved across the ports (∼10 mm). Some residual small oscillations were observed in 2/10 cortical neurons recorded. This suggests that a minority of cortical neurons measured were not connected efficiently to other parts of the circuit, so were less influenced by TTX application. Equally, as there is no direct connection between SNc or SNr and GP ports in the microfluidic device, the SN neuron response to TTX in the GP port must have depended on interconnectivity with striatal neurons residing in the middle port of the device. As before, we observed some residual spiking in the SNc port after application of TTX to the GP and striatal ports, likely due to the presence of pacemaker dopamine neurons that were not inhibited by the silencing of other neurons in the circuit.

## 5. Conclusions

This study was intended to fabricate an *in vitro* functional model to mimic a complex neuronal circuit. The fabricated device advances current state of the art *brain-on-a-chip* technology by demonstrating close co-localisation, directed connectivity and networked communication across a five-population neuronal circuit. The five-port microfluidic device design is inspired by the arrangement of *in vivo* BG circuitry, being seeded by primary neurons specifically isolated from various brain regions. Axonal outgrowth was directed from one port to an adjacent port via tapered micro-channels. Functional connectivity of the neuronal circuitry was studied using calcium imaging. The electrical activity of the circuitry was partly similar to what was expected from an *in vivo* basal circuitry, in that neurons both downstream and upstream of TTX application were silenced by this neurotoxin. This provided evidence that there is functional cellular connectivity across the neuronal populations cultured in this device. Some of the Ca^2+^ signalling data suggest that the neurons are likely still immature, and therefore excitatory in nature.

Further work is needed to develop a more mature neuronal circuit and refine methods for repeated long term functional measurements. Findings from the current study contribute to the body of work concerned with high throughput screening of potential therapies for neurodegenerative diseases such as Huntington’s and Parkinson’s diseases through the development and understanding of *in vitro* neuronal circuitry. Although there are still practical and functional issues to resolve in our system, future work examining the precise morphological structure and fabrication of a complex neuronal architecture with network function poses the exciting possibility of highly advanced *in vitro* systems. These will offer unprecedented insight into the development of neural tissues and reduction in function during disease. We expect adoption of this technology to further inform pharmaceutical and cellular testing aligned to the 3Rs (reduction, refinement and replacement of animal-based research) whilst also offering potential to become useful for pre-clinical evaluation of stem cell therapies.

## Access to research data

Data underlying this research is available on request from the authors. Mask designs for the 2-port NeuroChip can be found at our online repository [43] https://doi.org/10.17028/rd.lboro.8203175.
